# Miniaturized cardiopulmonary bypass: the Hammersmith technique

**DOI:** 10.1186/1749-8090-8-143

**Published:** 2013-06-03

**Authors:** Aziz Momin, Mansour Sharabiani, John Mulholland, Gemma Yarham, Barnaby Reeves, Jon Anderson, Gianni Angelini

**Affiliations:** 1 Cardiac Surgery and Clinical Perfusion, Imperial College London, London, UK; 2 Hammersmith Hospital, Imperial College London, London, UK

**Keywords:** Mini CPB, Conventional CPB, Heart surgery, Mini-Cardiopulmonary, Bypass

## Abstract

**Background:**

Conventional Cardiopulmonary Bypass (cCPB) is a trigger of systemic inflammatory reactions, hemodilution, coagulopathy, and organ failure. Miniaturised Cardiopulmonary Bypass (mCPB) has the potential to reduce these deleterious effects. Here, we describe our standardised ‘Hammersmith’ mCPB technique, used in all types of adult cardiac operations including major aortic surgery.

**Methods:**

The use of mCPB remains limited by the diversity of technologies which range from extremely complex, micro systems to ones very similar to cCPB. Our approach is designed around the principle of balancing the benefits of miniaturisation; reducing foreign surface area while maintaining patient safety.

**Results:**

From January 2010 to March 2011, a single surgeon performed 184 consecutive operations (Euro score Logistic 8.4+/-9.9): 61 aortic valve replacements, 78 CABGs, 25 aortic valve replacement and CABG and 17 other procedures (major aortic surgery, re-do operations or double/triple valve replacements).

Our clinical experience suggests that:

i. Venous drainage is optimally maintained using kinetic energy.

ii. Venous collapse pressure depends on the patient’s anatomy and cannula size, but most importantly on the negative pressure generated by venous drainage.

iii. The patient-prime interaction is optimised with antegrade and retrograde autologous priming, which mixes the blood and prime away from the tissues and results in a reduced oncotic destabilization.

iv. mCPB is a safe and reproducible technique

**Conclusion:**

The Hammersmith mCPB is a “next generation” system which uses standard commercially available components. It aims to maintain safety margin and the benefit of miniaturised system whilst reducing the human factor demands.

## Background

Conventional cardiopulmonary bypass (cCPB) remains the most common type of cardiopulmonary bypass (CPB), with only 4-10% of operations using miniaturised CPB (mCPB). Potential advantages of mCPB include a reduction in the deleterious effects of CPB, patient derived volume addition, and better physiological compatibility.

An early, small-randomized controlled trial (RCT) [1] reported that mCPB “marginally” reduced coagulation and inflammatory markers but also expressed concern about safety. Recently, RCTs have consistently reported benefits of mCPB, including reductions in length of stay in the intensive care unit, lower blood loss and transfusion requirements, improved renal and neurological outcomes [2-5], a higher mean arterial pressure during CPB, a lower consumption of vasoactive drugs, and a reduced inflammatory response. A meta-analysis of 33 RCTs showed mCPB to be associated with a lower risk of blood loss, postoperative stroke, and mortality compared to cCPB [6]. The applicability of these findings was limited by the diversity of mCPB technologies evaluated in the RCTs, which ranged from extremely complex, micro systems to ones very similar to cCPB technology.

Some of the diversity is explained firstly by advances in mCPB technology, which have improved the safety of mCPB [4,7], but also the absence of an agreed definition for the technology which allows many different systems to be included under the miniaturized umbrella. It remains a major challenge to standardise an optimal system capable of eliciting the benefits of miniaturisation while retaining an adequate safety margin. In this paper, we describe our Hammersmith mCPB system initially used by a single surgeon (JA) and now progressively adopted by the rest of the unit surgeons.

## Methods

Anaesthesia is maintained using a mixture of propofol and remifentanil. Blood gas analysis and activated clotting time (ACT) are checked regularly to maintain a minimum ACT of 400 seconds during CPB. Patients are cooled to 32°C. A cardiac index of 1.8 - 2.4 L/m^2^/min is used to determine each patient’s target normothermic cardiac output. Myocardial protection is achieved using either cold (4:1 ratio) blood-cardioplegia or intermittent cross-clamp fibrillation.

**Figure 1:**
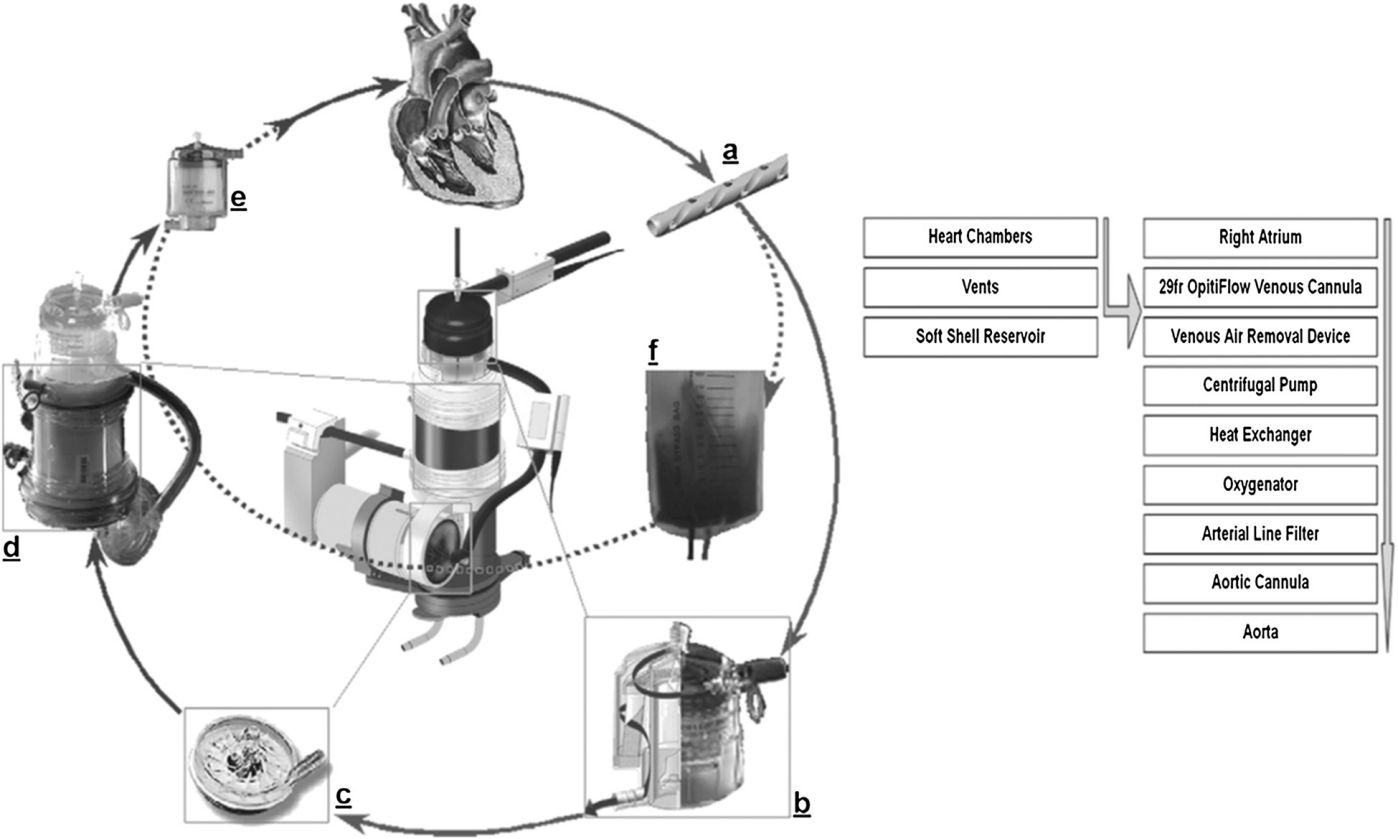
**Schematic and flowchart of the Hammersmith mCPB system**. **(a) **29-French OptiFlow venous cannula (Sorin Group, Mirandola, Italy); **(b) **venous air removal device; **(c)** centrifugal pump (Revolution Cardiopulmonary Bypass; Stöckert, Munchen, Germany); **(d) **heat exchange, and oxygenator module (Eos [Sorin Group, Mirandola, Italy]); **(e)** arterial line filter; and **(f) **parallel soft-shell reservoir.

Our mCPB system (Sorin Group Italia) is built from a set of standard components (Table 1) tailored to specific circumstances, i.e. the patients’ characteristics, planned procedure, and the perfusionist and surgical teams experience (Figure 1).

**Table 1:** Components of the standard Hammersmith mCPB circuit

**Prime Volume**	**134 ml (with ante grade and retrograde priming)**
**Foreign surface area**	Oxygenator – 1.1 m^2 *^
	VARD – 0.0105 m^2 *^
	Soft Shell Bag 0.0860 m^2 ^*or Midi Card – 0.0535 m^2^ *
	Total – 1.301 m^2 ^(1.268 m^2^)
	Tubing – 0.0747 m^2^
	Arterial line Filter – 0.0300 m^2 ^(D733, Low Prime Sorin group)
	Pre Bypass Filter (0.5 μm filter within the arterial-venous sash (Siever, [Sorin group])
	3/8 Arterial and Venous lines
**Air interface**	100 ml in Midi Card – 8.14 × 10^-4^ m^2^ *
**Air handling**	Air free venting as standard
	2× ¼ polyvinylchloride lines to act as an aortic root and Left Ventricular
	No air in soft shell bag
	Continuous communication with surgeon
**Safety features**	VARD *
	Ramp Down function
	Electronic clamp
**Cell saving device**	Dideco Electra [Sorin group, Italy]

* See Figure 1 for details; *VARD* Venous Air Removal Device.

We balance Foreign Surface Area (FSA) of the system with clinical applicability to maintain patient safety (Table 2). For example, we monitor and control the negative pressure in the venous line using the patient-specific venous collapse pressure as the threshold value. The venous cannula we use (Figure 1a) facilitates optimisation in this area of our system as its design increases the patient-specific venous collapse pressure.

**Table 2:** Advantages and disadvantages of mCPB and cCPB

		**mCPB**	**cCPB**
Venous cannula		29 Fr OptiFlow (Sorin)	34/46 Fr 2-stage (Medtronic)
	Advantage	• Multi-stage and longer length (increasing drainage and structural support in IVC)	• Convenient and easy placement
		• Less prone to collapse and blockage due to side holes and grooves	• This type of cannulae is used in common practice
	Disadvantage	• Rigid (requiring careful placement) as it extends further down the IVC	• Larger – increased risk of interaction with IVC/RA wall
			• 2 stage cannula less support in IVC thus more prone to collapse and decreased drainage from Hepatic veins and circulation
Venous line and drainage		3/8 inch tubing	½ inch tubing
	Advantage	• Smaller, active kinetic drainage	• Gravity syphon based
		• Monitored controlled drainage	• Simple, standard and convenient
		• Tailored to patient specific venous collapse pressure	
	Disadvantage		• Uncontrollable
			• Not routinely monitored
VARD		Advisable to use in mCPB but not compulsory	VARD is not required due to the presence of the Venous reservoir but it has been proven to be of benefit in all CPB circuits [8]. However, it is not commonly used.
	Advantage	• Enhances safety	• Cheaper
		• Efficient gross air removal	• Simple open system
		• Active micro air removal	• The reservoir filters and removes gross air easily
		• Decreases FSA versus standard filters used in CPB venous reservoir’s	• Continuity
		• All air introduction into system	
	Disadvantage	• Require perfusion experience	• No active removal of micro-embolic air (just passive)
		• Extra Component of circuit	• Venous reservoir in series (continued FSA exposure)
		• Vented blood has to be manually returned back into the systemic system	• Increases FSA
Reservoir*		SSR or Midi card	Venous reservoir (Sorin Evo)
	Advantage	• Closed (no ‘in series’ blood-air interface- limits FSA exposure)	• Open
		• Decreased damage to blood cells	• Common practice
		• Optimises vent management	• Venting possible
		• Midi card ‘in parallel’ automatic air removal	• Low Pressure Suction and blood venting possible
		•	• Vented blood is automatically returned to the systemic circulation
	Disadvantage	• SSR requires manual air bubble removal	• ‘In series’ Blood-air interface
		• No Low Pressure Suction (an issue in cases where there are high volumes of LPS)	• Damage to blood cells
		• Vented blood has to be manually returned back into the systemic circulation	• Disguises poor suction/vent management
Centrifugal pump		Revolution (Stöckert, Germany)	Standard roller pump
	Advantage	• Non-occlusive	• ½ inch silicon tubing
		• Pressure regulates	• Cost-effective
		• Gross safety mechanism	
		• Less blood cell trauma	
	Disadvantage	• Cost and training	• Occlusive (No pressure regulation)
Heat exchanger and oxygenator		Eos (Sorin Group, Italy)	Avant (Sorin Group, Italy)
	Advantage	• 1.1 m^2^ (decreased) FSA	• High ‘factor of safety’
		• Efficient use of fibre bundle capacity	• 7.5 L/min blood flow
		• high ratio of gas exchange surface area to FSA	
	Disadvantage	• Reduced (but acceptable) ‘factor of safety’	• 1.8 m^2^ FSA
			• Excessive ‘factor of safety’ for our patient population
Arterial line filter		Pall AL6 low prime	Pall AL6 low prime

* We begin training junior perfusionists with Midi card and after experience is gained, we move to SSR for routine cases. With further experience both by surgical and perfusionist teams, we move forward to Soft Shell Reservoir (*SSR*) for all cases regardless of complexity. The characteristics of these two options are described below.

A venous air removal device (VARD) facilitates the management of gross and microscopic air removal, which is a key safety advantage (Figure 1b) and provides ‘active’ microscopic air removal, rather than the ‘passive’ micro air removal achieved using a cCPB hard-shell venous reservoir. We prefer a centrifugal pump although a standard roller pump can also be used as long as the pump is linked to the venous line pressure (Figure 1c). We carefully consider the size of FSA we expose the patients’ blood to, as well as the exposure time. For example we use an oxygenator with a very high ratio of gas exchange surface area to FSA. Manufacturers quote the gas exchange surface area but it is important to know how much FSA is required to achieve that gas exchange surface area. A well design oxygenator boasting good fluid dynamics will minimise the FSA required to provide the gas exchange surface. We use a 1.2 m^2^ oxygenator which provides a 1.1 m^2^ gas exchange surface (Figure 1d). The advantages of using a smaller 1.1 m^2^ gas exchange surface have to be balanced against reducing your ‘factor of safety’. This is the residual capacity of the oxygenator that is not used. In our experience, the margin remains acceptable even at normothermic levels where we would comfortably run FiO2’s at 75% (25% FoS) rather than at 65% (35%FoS). Versatility is enhanced by allowing for an optional isolated soft-shell reservoir (SSR) or Midi card running in parallel to the systemic circulation (Figure 1f and Table 3). This option facilitates management of circulating volume in high-risk circumstances, although at the expense of increased FSA.

**Table 3:** **mCPB**: **soft shell reservoir and Midi card options**

		**Options**	
		**Soft shell reservoir**	**Midi card**
Patient		All	All
Operation		AVR, CABG, AVR + CABG	Complex operation, redo, Aortic root, Mitral,
		Experienced perfusionist *	Case selection
Device	Parallel	Yes	Yes
	Blood-air interface	No	Yes (minimal)
Vented Blood	Air removal	Manual (active)	Automatic
			Open reservoir
			Independent of perfusionist
	Venting	Experienced management and good communication	Easily managed, not labour intensive

*AVR *Aortic Valve Replacement, *CABG *Coronary Artery Bypass Graft, *FSA *Foreign Surface Area, *IVC *Inferior Vena Cava, *SSR *Soft shell Reservoir, *VARD *Venous Air Removal Device. * We begin training junior perfusionists with Midi card and after experience is gained, we move to SSR for routine cases. With further experience both by surgical and perfusionist teams, we move forward to Soft Shell Reservoir (*SSR*) for all cases regardless of complexity. The characteristics of these two options are described below.

MCPB requires the patient to be used as a reservoir. Consequently, it can pose challenges, such as the need to reposition the operating table to facilitate venous drainage and filling. The use of a SSR eliminates this problem. A key feature of the SSR is its position in parallel, not in series as in cCPB, thus eliminating blood FSA exposure on every pass through the system (limiting exposure time). It has no air interface and the perfusionist manually removes any air introduced by the vent into the SSR. Therefore, a SSR can be routinely used in circumstances that are unlikely to give rise to complex air/vent management issues. For more complex circumstances, a hard shell reservoir (HSR), Midi Card, is used; despite a limited air interface, the Midi Card allows easier air management from the vent and suction because it is automated. Both of these aspects of our system can be perceived to be a disadvantage but when considered in context the advantages in terms of clinical applicability outweigh any disadvantages. Take for example the introduction of a soft-shell reservoir, clinical applicability is significantly increased, it also presents an additional FSA of 0.086 m^2^ which is insignificant compared to the FSA of an Oxygenator.

## Results

Review of our experience using mCPB was approved by the clinical audit committee of the Imperial College National Health Service Trust to meet ethical and legal requirements, and individual consent was waived. From January 2010 to March 2011, a single surgeon (JA) carried out 61 isolated aortic valve replacements, 78 CABGs, 25 aortic valve replacement and CABG and 17 other operations (major aortic surgery, re-do operations or double/triple valve replacements) on 184 consecutive patients. The mean logistic Euro score was 8.4 +/- 9.9 SD. The consultant performed 74% of operations and surgical residents performed the remainder under supervision.

**Figure 2:**
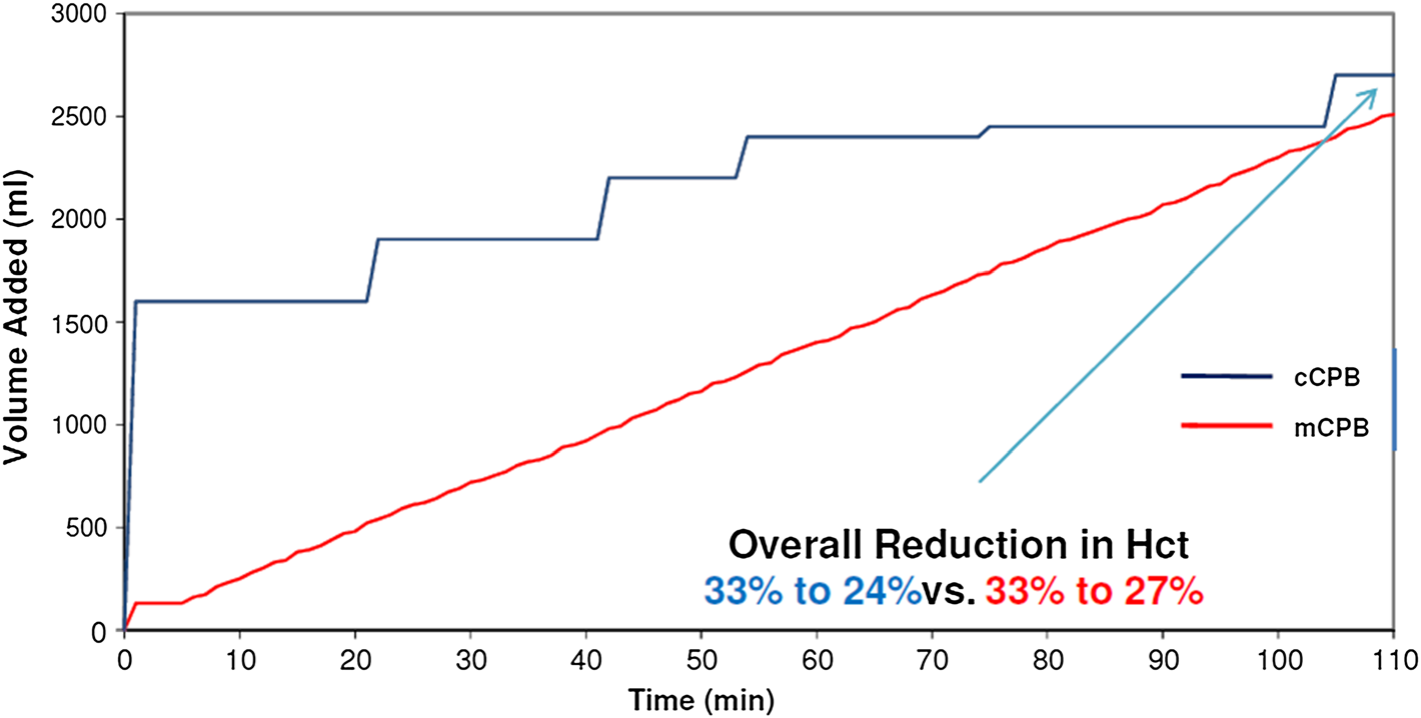
**Haematocrit stabilisation mCPB vs. cCPB. **Graphs are illustrative only; each panel is based on data for a randomly selected individual per technique: Volume Management.

**Figure 3:**
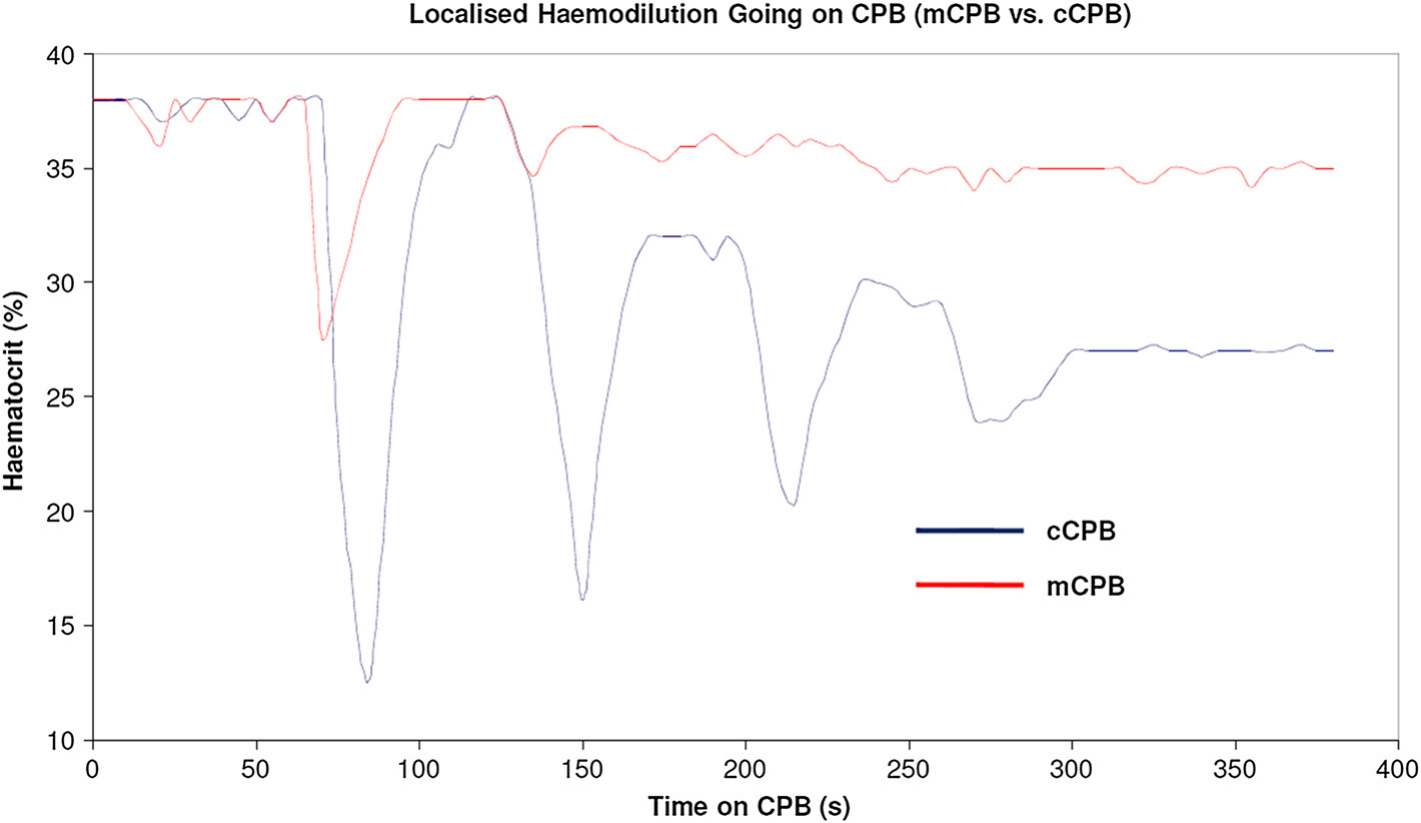
**Haematocrit stabilisation mCPB vs. cCPB. **Graphs are illustrative only; each panel is based on data for a randomly selected individual per technique: Patient/Prime Interaction.

Our clinical experience has led us to the following conclusion:

Venous drainage is optimally maintained using kinetic energy which provides controlled removal of fluid volume from the heart (controlled negative pressure), a benefit that is rarely discussed. We run a pressure isolator from the venous line that is connected to an electrical pressure monitor on the heart lung machine. This monitoring provides us with a digital readout of the pressure in the venous line. Accurately monitoring and controlling this quality indicator facilitates optimisation of the haemodynamic balance between circulating volume, cardiac output (arterial pump flow) and SVR.

i. Venous collapse pressure is patient-specific. It depends on the patient’s anatomy and cannula size, but most importantly on the negative pressure generated by venous drainage. In a pilot study of 20 patients we found the patient-specific venous collapse pressure varies from -8 mmHg to -54 mmHg. The same study showed that gravity venous drainage generates a range of -26 mmHg to -48 mmHg and vacuum assisted generates a range of -56 mmHg to -79 mmHg. This collapse pressure varies from -8 mmHg to -54 mmHg. Gravity venous drainage generates a range of -26 mmHg to -48 mmHg and vacuum assisted venous drainage generates a range of -56 mmHg to -79 mmHg. Given that CPB most commonly utilises gravity venous drainage (uncontrolled and unmonitored), the negative pressures generated will exceed the patient specific collapse pressure for a large group of patients in our pilot. This pressure magnitude can cause venous collapse and even though it may not stop blood drainage to the circuit, the risk of venous congestion (especially Liver) and resulting sub-optimal end organ perfusion is increased. Vacuum assisted venous drainage increases this risk further. The benefits of accurately monitored and controlled kinetic venous drainage provided by mCPB will minimise this risk.

ii. The patient-prime interaction is optimised with ante grade and retrograde autologous priming. This method mixes the blood and prime away from the tissues and results in a reduced oncotic destabilization. As a result mCPB stabilises haematocrit much quicker, whereas the initial prime load for cCPB requires several passes through the circuit to stabilise the haematocrit (Figures 2 and 3).

Ti *et al*. [9] reported cardiac index targets were difficult to achieve using mCPB. This can happen if the management of the technology is not considered, resulting in volume restrictive strategy focusing on maintaining haematocrit only. Our philosophy is to shift attention to the human management aspect of the technology, optimising patient-volume interaction, haematocrit, arterial flow and circulating volume without being volume restrictive. The mCPB closed system allows better informed haemodynamic balance management. Figure 2A shows the benefit in haematocrit management of gradual and pre-emptive volume addition, with the volume determined by the patient’s requirement. A low haematocrit does not entail a failure of the technology if the hemodynamic balance is managed accurately and the patient requires volume, all the other advantages outlined still remain. Nevertheless, there is no doubt that mCPB places greater demands on the perfusionist, as it requires active, informed management throughout the operation in order to integrate optimally drug/fluid addition, blood suction, vent and SVR management.

The patient cohort described was completely unselected, with no exclusions due to the technical complexity of the operation. Table 4 describes the patient’s preoperative, operative and postoperative characteristics. Less than 9% of patients had red blood cells transfused, and less than 5% of patients required platelets or fresh frozen plasma. The frequencies of post-operative complications were unremarkable given the nature of the cohort. No technical problems related to the use of the mCPB were reported throughout the all series.

**Table 4:** **Pre**, **intra and post**-**operative characteristics** (**n** = **184**)

**Patient data**		**Mini CPB (n. =180)**	
		**n**	**%**
*Male*		130	70.7
*Euro score *(*logistic*)		8.4 (±9.9)	
*Previous myocardial infarction*		44	23.9
*Diabetes*		58	31.5
*Hypertension*		155	84.2
*Preoperative renal disease*	Creatinine >200 umol/l	13	7.1
	Creatinine >200 umol/l and dialysis	2	1.1
	Dialysis for CRF >6 weeks prior to surgery	3	1.6
*Extent of coronary disease*	One vessel	15	8.2
	Two vessels	20	10.9
	Three vessels	63	34.2
*Ejection fraction category*	Fair (LVEF 30-49%)	24	13.0
	Poor (LVEF <30%)	17	9.2
*Cross*-*clamp time*, (*minutes*)			42.9 (±29.5)
*CPB time*, *minutes*			71.0 (± 41.9)
*Intra*-*aortic balloon pump*		4	2.2
*Blood product usage*	PRC	8	4.3
	PRC ,FFP, platelets	5	2.7
*Total chest tube drainage *(*ml*)		801 (±483)	
*Reoperation for bleeding*		2	1.1
*Renal Complications*		3	1.6
*Stroke*		1	0.5
*In hospital death *(*30 days*)		2	1.1

*CRF *Chronic Renal Failure, *LVEF *Left Ventricular Ejection Fraction, *CPB *Cardiopulmonary Bypass, *FFP *Fresh Frozen Plasma.

Mean and standard deviation.

## Discussion

Miniaturised CPB should be viewed as a delicate balance between benefit (for the patient and publicly funded health services), intra-operative safety margins and clinical applicability rather than as a strategy to minimize the circuit without clear objectives. A common sense and informed approach needs to be applied when considering what to minimise; For example, switching from a 1.8 m^2^ to a 1.1 m^2^ oxygenator (where the latter is more efficient in terms of gas exchange) when compared to a more common strategy used by perfusion teams to limit FSA, it equates to 42 m of ^3^/_8_” tubing. Our choice of oxygenator facilitates a substantial reduction in FSA with an acceptable safety margin and 4.5 litre flow per minute as compared to a traditional Avant Sorin oxygenator which has 1.8 m^2^ FSA with an inefficient use of fibre bundles despite a relatively higher safety margin.

mCPB requires active participation, awareness and increased technical knowledge of the cardiac team. With such involvement, mCPB enhances monitoring and consequently reduces introduction of air from sample lines, ports, the surgical field and additions of fluid. It also brings to the attention of the perfusionist poor vent and suction management, encourages good multidisciplinary communication, and promotes continuous monitoring of SVR and hemodynamic balance. Highlighting sub-optimal CPB management improves quality. Emphasising the introduction of air from sample ports and fluid additions lead to a decrease in air emboli and may reduce the risk of a neurological complication. Venting is important to provide a bloodless field with optimal vision; it should be balanced with the resultant damage caused to the blood constituents. mCPB promotes optimisation of this balance. All pericardial suction blood is processed via cell saving technology.

Before undertaking more complex cases with mCPB, perfusionists should acquire the skill set to manage mCPB in low risk circumstances because it takes time to learn to manage mCPB technology to maximise its benefits. However, we believe the greatest benefits of mCPB may arise for more complex operations and higher risk patient populations with significant co-morbidity. Despite evidence that mCPB has important clinical benefits [6] it has not been widely implemented, probably because previous RCTs evaluated diverse systems several of which had limited clinical applicability to usual care.

After initial development of our mCPB technique and implementation in usual care by one surgeon, it is now progressively adopted by the rest of the departmental surgeons.

## Conclusion

The Hammersmith mCPB is a “next generation” system which uses standard commercially available components. It aims to maintain safety margin and the benefit of miniaturised system whilst reducing the human factor demands.

## Competing interest

The authors declare that they have no competing interests.

## Authors’ contributions

AM designed the study and drafted the manuscript; MS analysed the data, contributed to the study design and drafting the manuscript; JM was the main contributor to developing the mCPB; GY helped designing the system as a perfusionist; BR contributed to drafting the manuscript; JA was the main user of the system as a surgeon; GA contributed to drafting the paper. All authors read and approved the final manuscript.
